# Single-Dose Toxicity Study of Self-Assembling A6K/Sodium Borocaptate (BSH) Peptide Nanotubes as a New Boron Delivery Agent for Boron Neutron Capture Therapy (BNCT) in Mice

**DOI:** 10.3390/cancers18091382

**Published:** 2026-04-27

**Authors:** Miharu Kano, Katsuaki Ieguchi, Tomonari Kasai, Kazuki Tsuchida, Yosuke Sasaki, Eisuke Shiozawa, Kouzou Murakami, Yasuaki Ichikawa, Satoshi Wada, Naoki Hayashi, Toshiko Yamochi

**Affiliations:** 1 Department of Pathology, School of Medicine, Showa Medical University, 1-5-8 Hatanodai, Shinagawa-ku, Tokyo 142-8555, Japan; 2 Division of Breast Surgical Oncology, Department of Surgery, School of Medicine, Showa Medical University, 1-5-8 Hatanodai, Shinagawa-ku, Tokyo 142-8555, Japan; 3 Center for Electron Microscopy, Showa Medical University, 1-5-8 Hatanodai, Shinagawa-ku, Tokyo 142-8555, Japan; 4 Energy System Engineering Research Group, Department of Applied Energy, Graduate School of Engineering, Nagoya University, Furo-cho, Chikusa-ku, Nagoya 464-8603, Japan; 5 Division of Radiology, Department of Radiology, School of Medicine, Showa Medical University, 1-5-8 Hatanodai, Shinagawa-ku, Tokyo 142-8555, Japan; 6 Department of Clinical Diagnostic Oncology, Clinical Research Institute for Clinical Pharmacology and Therapeutics, Showa Medical University, 1-5-8 Hatanodai, Shinagawa-ku, Tokyo 142-8555, Japan

**Keywords:** boron neutron capture therapy, boron delivery, acute toxicity, toxicity study, preclinical study

## Abstract

**Background**: Boron neutron capture therapy (BNCT) is a type of targeted radiotherapy with considerable therapeutic potential that may be combined with immune checkpoint inhibitors (ICIs) to enhance systemic antitumor immunity. Its efficacy relies on the efficient and safe delivery of boron to cancer cells. This study evaluated the acute toxicity of a self-assembling peptide-based boron carrier composed of A6K and sodium borocaptate (BSH) at a 1:10 molar ratio (A6K/BSH boron drug), which had previously shown excellent tumor-selective accumulation and prolonged intracellular retention. **Methods**: A single-dose intraperitoneal toxicity study was performed in 6-week-old BALB/c mice (n = 6 per group; 3 males and 3 females). Animals received BSH-equivalent doses of 0, 30, 100, 300, or 600 mg/kg and were observed for 14 days. Mortality, clinical signs, body weight, gross necropsy findings, and histopathological characteristics of major organs were then assessed. **Results**: No mortality or treatment-related clinical signs were observed. Body weight changes were comparable between the control and treated groups. Gross necropsy revealed no treatment-related abnormalities. Histopathology showed mild hepatocellular hypertrophy and granular degeneration without dose dependency. No other organ toxicities or sex-related differences were detected. **Conclusions**: A single intraperitoneal administration of the A6K/BSH boron drug up to 600 mg/kg (BSH-equivalent) produced no evident acute systemic toxicity, suggesting that the approximate lethal dose exceeds 600 mg/kg for both sexes. This initial safety assessment supports the further development of the A6K/BSH boron drug as a boron delivery agent for BNCT. Further studies are needed to confirm its safety under clinically relevant conditions.

## Simple Summary

Boron neutron capture therapy (BNCT) is type of a targeted radiotherapy that selectively kills tumor cells and enhances systemic antitumor effects when combined with immune checkpoint inhibitors (ICIs). The success of this therapy relies on the efficient delivery of boron to cancer cells. We developed a peptide-based boron carrier comprising A6K and sodium borocaptate (BSH) at a 1:10 molar ratio, which enhances tumor-specific accumulation and boron retention. Acute A6K/BSH boron drug toxicity was evaluated in mice. BALB/c mice received a single intraperitoneal dose of 0, 30, 100, 300, or 600 mg/kg (BSH-equivalent) and were monitored for 14 days. No mortality or overt toxicity was observed. Body weight, necropsy, and organ histopathology showed only mild, non-dose-dependent liver changes, with no other organ effects. These findings indicate that A6K/BSH boron drug is well tolerated and support its further development for BNCT. However, additional studies are needed to confirm its safety.

## 1. Introduction

Boron neutron capture therapy (BNCT) is a type of targeted radiotherapy based on the nuclear capture and fission reaction between boron-10 (^10^B) and thermal neutrons [1]. After administration of a ^10^B-containing compound, boron is expected to preferentially accumulate in tumor tissues [2]. Subsequent neutron irradiation induces the ^10^B (n, α) ^7^Li reaction, which generates high-linear energy transfer (high-LET) particles (α particles and recoiling ^7^Li nuclei) that can kill tumor cells [3]. Considering that the ranges of these particles are same order of a single cell diameter (approximately 5–10 μm), radiation damage is largely confined to boron-containing cells, thereby sparing the surrounding normal tissues [3]. This unique physical property enables highly localized cancer treatment [4]. Aside from its direct cytotoxic effects, BNCT may also promote systemic antitumor immune responses, occasionally inducing the abscopal effect characterized by regression of non-irradiated distant tumors. Despite the rarity of this phenomenon, accumulating evidence suggests that its frequency can increase in the presence of immune checkpoint inhibitors (ICIs), such as anti-PD-1/PD-L1 or anti-CTLA-4 antibodies [5,6,7]. Given that BNCT delivers highly localized high-LET radiation at the cellular level, it may represent a promising modality for combination with ICIs to enhance systemic antitumor immunity.

Despite its therapeutic potential, BNCT faces several challenges, with the development and optimization of boron delivery agents being a key issue [1,8,9]. At present, two agents have been clinically used for BNCT, namely sodium borocaptate (BSH; Na_2_B_12_H_11_SH) and L-4-boronophenylalanine (BPA) [10]. However, both agents have their limitations, including variable tumor uptake, heterogeneous intratumoral distribution, and insufficient tumor retention [10]. To address these issues, various boron carriers have been proposed [11,12]. Among them, self-assembling A6K peptide nanotubes formulated with BSH at a defined molar ratio (A6K:BSH = 1:10, mol/mol) have been developed as a novel boron delivery system [13].

A6K is a short peptide composed of six alanine residues and one lysine residue (sequence: AAAAAAK). It spontaneously self-assembles in an aqueous solution to form supramolecular structures, including nanofibers and nanotubes [14]. This self-assembly is primarily driven by hydrophobic interactions among alanine residues and β-sheet formation, whereas the positively charged lysine residue contributes to structural stabilization and aqueous solubility [15,16,17]. Studies have also identified A6K as a promising drug carrier that can enhance the solubility of hydrophobic drugs and improve intracellular delivery without increasing cytotoxicity [18]. More broadly, self-assembled peptide nanostructures possess characteristics that favor drug delivery, including biocompatibility, biodegradability, and low immunogenicity [19,20,21]. By leveraging these properties, A6K can encapsulate boron-containing compounds, such as BSH, forming peptide-functionalized nanotubes (hereafter referred to as the “A6K/BSH boron drug”) that retain the self-assembled architecture of A6K. These nanotubes have been shown to facilitate tumor-selective boron delivery and intracellular retention in cancer cells for over 12 h [13].

Despite the potential utility of the A6K/BSH boron drug in terms of improved tumor-selective accumulation and intracellular retention of boron, its preclinical safety profile has remained insufficiently characterized. Importantly, although peptide-based nanostructures have been broadly studied as drug delivery platforms and their general biocompatibility is well documented [19,20,21], these previous findings may not fully predict the safety profile of the present formulation. First, the biological behavior of boron compounds may be altered by encapsulation within self-assembling peptide carriers [13]. Second, the nanotubular structure formed by A6K/BSH differs physicochemically from other particulate delivery systems and may therefore influence in vivo distribution and tissue interactions differently [13]. Third, because BNCT requires administration at relatively high boron concentrations [1,2,3,4], safety data obtained under conventional low-dose nanocarrier conditions may not adequately predict toxicity under clinically relevant exposure levels. Therefore, an independent safety evaluation of the A6K/BSH boron drug is necessary.

Accordingly, the present study aimed to evaluate the acute toxicity of the A6K/BSH boron drug in mice using a single-dose toxicity study to provide initial safety data supporting its further development for BNCT.

## 2. Materials and Methods

### 2.1. Study Approval

All animal procedures were approved by the Animal Care and Use Committee of Showa Medical University (approval No. 125069) and were performed in accordance with the institutional regulations and the Act on Welfare and Management of Animals (Japan).

### 2.2. Test Substance

The A6K/BSH boron drug was prepared as previously described [13]. The A6K peptide was kindly provided by 3-D Matrix, Ltd. (Tokyo, Japan), whereas BSH was purchased from KATCHEM (Prague, Czech Republic). A6K was dissolved in distilled water (Otsuka Pharmaceutical Co., Ltd., Tokyo, Japan) to prepare solutions of 2–20 mM, whereas BSH was dissolved in distilled water to prepare solutions of 20–200 mM. Both solutions were mixed at a defined molar ratio of A6K:BSH = 1:10 to obtain four formulations with final concentrations of 2:20, 5:50, 10:100, and 20:200 mM (A6K mM:BSH mM). Table 1 details the amount of BSH contained in each formulation. All formulations were prepared immediately prior to administration.

Previous reports demonstrated that A6K/BSH prepared at an A6K:BSH molar ratio of 1:10 forms spherical particles when observed under an electron microscope [13]. In the present study, the A6K/BSH boron drug was prepared using the same method; however, particle size distribution, aggregation state, and nanostructure stability were not re-evaluated. This approach was adopted because the formulation was prepared using the identical protocol described previously [13], and the primary objective of the present study was to evaluate systemic toxicity rather than to perform physicochemical characterization. Nevertheless, the absence of confirmatory characterization data for the specific batches used in this study is acknowledged as a limitation.

### 2.3. Experimental Animals

Six-week-old BALB/c mice (male and female) were purchased from CLEA Japan, Inc. (Tokyo, Japan). Mice were housed under controlled conditions (temperature 20–23 °C; relative humidity, 45–50%; 12 h light/dark cycle; ventilation, 6–15 air changes per hour) with ad libitum access to food and water. To minimize potential confounding effects, animals were housed under identical environmental conditions, and all procedures were conducted in a consistent manner across groups. No specific measures, such as randomization of treatment order or cage location, were implemented. All animals were acclimatized for 1 week before A6K/BSH boron drug administration. Only animals showing no abnormal findings during acclimation were enrolled in the study. This inclusion criterion was established a priori. No additional exclusion criteria were predefined for the experimental phase or data analysis.

### 2.4. Experimental Design

A total of 30 mice were used in this study. Mice were randomly allocated (n = 3 males and n = 3 females per group) to five groups according to the target BSH dose (mg/kg body weight): G0, 0 (control); G1, 30 mg/kg; G2, 100 mg/kg; G3, 300 mg/kg; and G4, 600 mg/kg. Group assignment was performed in a non-systematic manner at the start of the experiment. The sample size was determined based on conventions commonly used in exploratory single-dose toxicity studies and in consideration of animal welfare to minimize the number of animals used. No formal a priori sample size calculation was performed. The control group received sterile saline (400 μL/mouse; Otsuka Pharmaceutical Co., Ltd., Tokyo, Japan). Dose levels were selected with reference to a previously reported effective dose of BSH 42.0 mg/kg [13] and a preliminary report indicating lethality at 600 mg/kg for BSH alone [22]. Although no independent dose-finding data were available for the A6K peptide carrier itself, A6K consists exclusively of natural amino acids (six alanine and one lysine residues) and may undergo proteolytic degradation under physiological conditions [14,15]. Moreover, prior in vitro studies demonstrated no apparent cytotoxicity of the A6K/BSH boron drug at the concentrations tested [13], supporting the selection of BSH-based dose levels as a reasonable starting point for this initial toxicity evaluation. Volumes administered to each group were calculated based on the BSH content of each formulation (Table 1) and are summarized in Table 2.

Although the A6K/BSH boron drug is intended to be clinically administered via the intravenous route, the high-concentration formulation used in this study was highly viscous and could not be passed through a 0.2-μm syringe filter. Such poor filterability raises concerns regarding the presence of aggregates or microbubbles, which could increase the risk of acute embolism upon intravenous injection [23,24]. Therefore, to minimize unnecessary lethal events and ensure animal welfare, this single-dose toxicity study administered the test substance intraperitoneally rather than intravenously.

No blinding was implemented at any stage of the study. Investigators were aware of the group allocation during allocation, conduct of the experiment, outcome assessment, and data analysis.

### 2.5. Animal Observation

Animals were closely monitored on the day of dosing (day 0) at 0.5, 1, 2, 4, and 6 h after administration. Thereafter, they were observed once daily from day 1 through day 14. Clinical observations included mortality and signs of toxicity, such as abnormal respiration, abnormal gait/posture, tremor, piloerection, and increased or decreased activity. The time of onset and recovery (if applicable) were recorded throughout the observation period. Body weight was measured on day 0 (before dosing) and on days 3, 7, and 14 after administration.

### 2.6. Necropsy and Histopathological Evaluation

Any animal found dead during the observation period was necropsied within 24 h. All surviving mice were euthanized on day 14, after which a complete gross necropsy was performed with recording of macroscopic findings. Tissues from the brain, thymus, heart, lungs, liver, pancreas, spleen, kidneys, stomach, small intestine, colon, bone marrow (femoral bone), eyeballs, submandibular gland, testes, ovaries, and uterus were collected for histopathology. All samples were fixed in 4% buffered formaldehyde for histopathological analysis. The tissues were dehydrated through a graded ethanol series and embedded in paraffin. Paraffin-embedded tissues were sectioned at a thickness of 4 μm using a blade (Feather Microtome Blade A35; Feather Safety Razor Co., Ltd., Osaka, Japan). Sections were deparaffinized in xylene, rehydrated through graded ethanol, and stained with hematoxylin and eosin. Whole-slide images were acquired using a digital slide scanner (NanoZoomer S60v2, Hamamatsu Photonics K.K., Hamamatsu, Japan) and analyzed with NDP.view 2.9.29 software (Hamamatsu Photonics K.K., Hamamatsu, Japan).

### 2.7. Statistical Analysis

Body weight changes in each group were summarized and presented as mean ± standard deviation. Comparisons between the control and each treatment group were performed using Steel’s multiple comparison test. Analyses were conducted separately for males and females. Statistical analyses were performed using Python (version 3.11.2) (Python Software Foundation, Wilmington, DE, USA), with a two-sided *p* value of <0.05 indicating statistical significance.

## 3. Results

No animals, experimental units, or data points were excluded from the analysis. All animals allocated to each group were included in the final analysis. For all analyses, the sample size was n = 6 per group (3 males and 3 females).

### 3.1. Mortality and Clinical Observations

No mortality was observed in any group throughout the observation period. Furthermore, neither the observations conducted on the day of administration nor the subsequent daily examinations revealed any signs of systemic toxicity, including abnormal respiration, abnormal gait/posture, tremor, piloerection, and increased or decreased activity (Table 3).

### 3.2. Body Weight Changes

**Figure 1:**
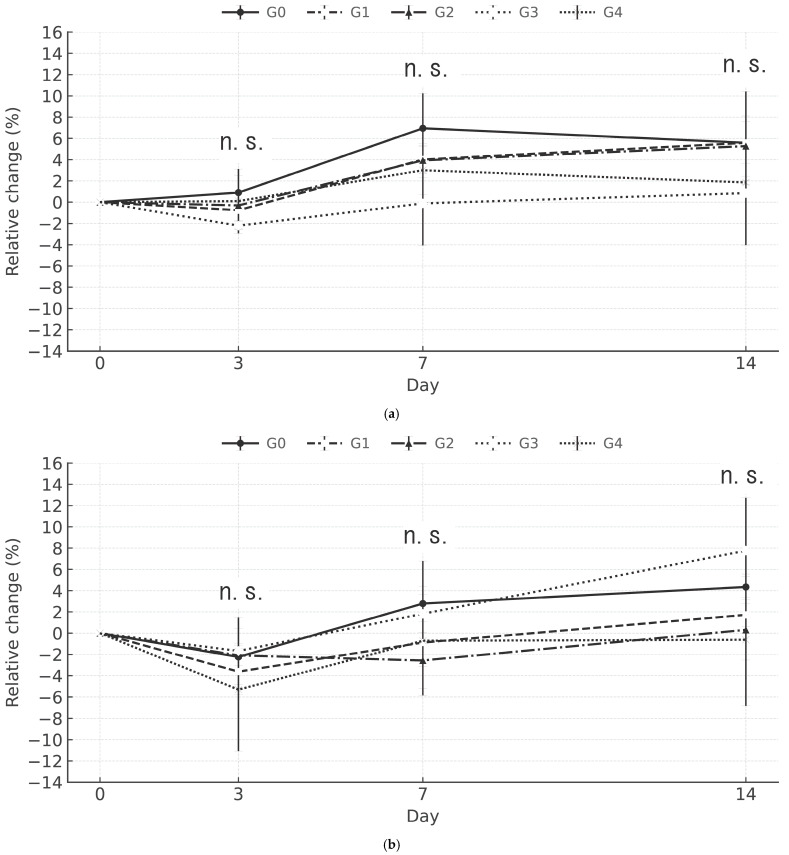
Relative change (%) in the body weight of male (**a**) and female (**b**) mice. G0/0 represents the control group, whereas G1/30, G2/100, G3/300, and G4/600 represent the groups receiving the indicated doses of the A6K/BSH boron drug. The x-axis shows the number of days after injection. No significant differences between the experimental and control groups were observed using Steel’s multiple comparison test. n.s., not significant.

Changes in body weight over time are presented in Figure 1a,b for male and female mice, respectively. All A6K/BSH boron drug groups (i.e., G1/30, G2/100, G3/300, and G4/600) showed body weight changes comparable to that in the G0/0 group. No significant differences (*p* > 0.05) in the bodyweight changes were observed among the male or female groups throughout the study.

### 3.3. Macroscopic Findings on Necropsy

Visual observation upon necropsy revealed no abnormal findings associated with the test substance in any of the experimental groups (Table 4).

### 3.4. Histopathological Evaluation

**Figure 2:**
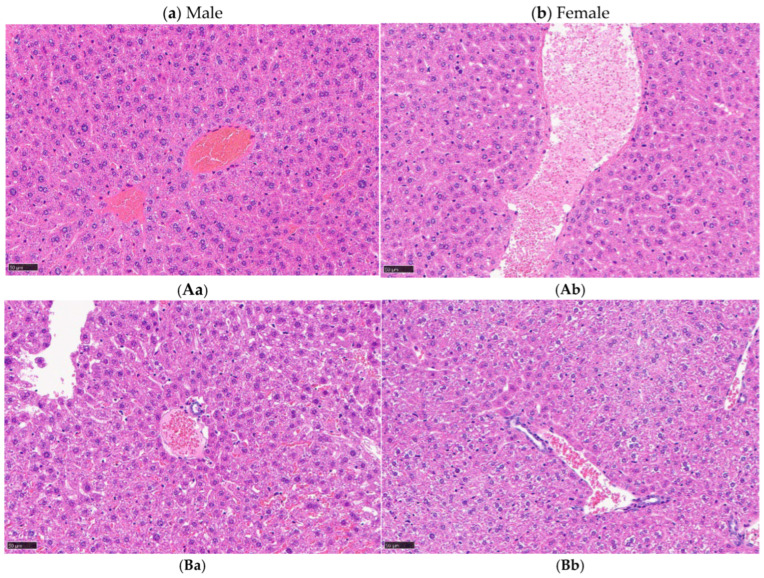

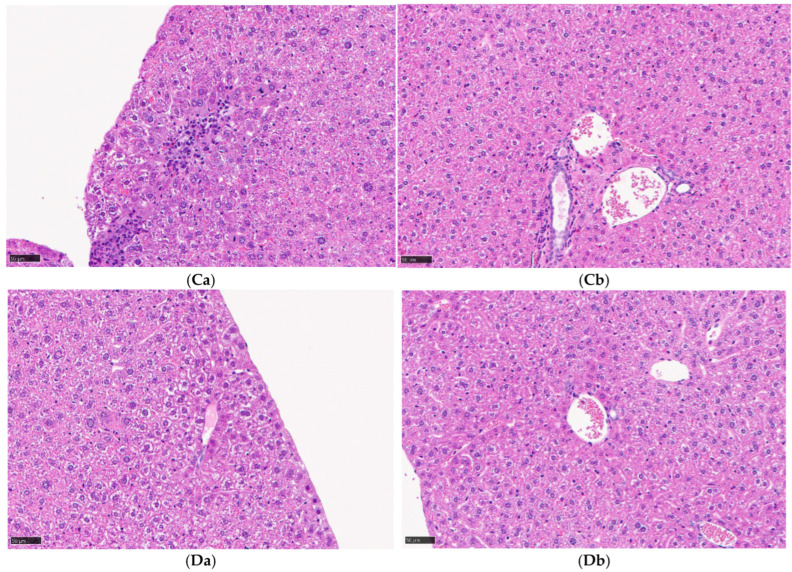

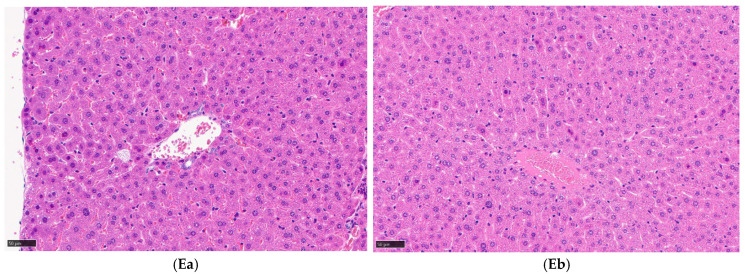
Histopathology of the liver. G0/0 indicates the control group in males (**Aa**) and females (**Ab**). Mild hepatocellular hypertrophy and granular degeneration were observed in the G1/30 (**Ba**,**Bb**), G2/100 (**Ca**,**Cb**), G3/300 (**Da**,**Db**), and G4/600 (**Ea**,**Eb**) groups in both males and females.

Liver histopathology revealed mild hepatocellular hypertrophy and granular degeneration in all males and females in the G1/30, G2/100, G3/300, and G4/600 groups but not in the control group (G0/0). No sex-related differences were noted. Furthermore, no apparent dose-dependent differences were identified among the treated groups. Histopathological analyses of the other organs revealed no abnormalities. Representative images and incidences of the findings are presented in Figure 2 and Table 5, respectively. Detailed histopathological findings for other major organs evaluated (heart, lungs, pancreas, kidneys, and bone marrow), none of which showed abnormalities, are presented in Supplementary Table S1.

## 4. Discussion

A single-dose toxicity study involving the intraperitoneal administration of self-assembling A6K/BSH peptide nanotubes (A6K/BSH boron drug) in mice at doses up to 600 mg/kg (expressed as the BSH-equivalent dose) resulted in no mortality, no apparent clinical signs, and no gross pathological abnormalities. Furthermore, no significant loss of body weight was observed. Given that changes in body weight and feed intake in animals usually occur following substance-induced toxicity [25,26], the absence of such changes in the present study further supports the favorable safety profile of the A6K/BSH boron drug. Histopathological examination revealed mild hepatocellular hypertrophy and granular degeneration in the liver in all treated animals. Although these findings were considered treatment-related, they showed no clear dose dependency and were not accompanied by necrosis, inflammation, or fibrosis. According to previous reports, mild hepatocellular hypertrophy without progressive degenerative changes may represent an adaptive metabolic response rather than an adverse toxic effect [27,28]. Therefore, these hepatic changes were considered to be more consistent with a non-adverse adaptive response than with overt hepatotoxicity. Moreover, no apparent inflammatory changes, necrosis, or fibrosis were observed in the major organs, including the lungs and heart. Under the present experimental conditions, absent or minimal organ toxicity was observed.

Considering studies reporting sex-related differences in responses to toxicity for certain drugs [29,30], both male and female mice were evaluated in the current study. However, no sex-related differences in mortality, clinical signs, body weight changes, or gross pathological findings were observed. Furthermore, histopathological findings were comparable between males and females. Overall, these results suggest no sex-related differences in the toxicity profile of the A6K/BSH boron drug under the present experimental conditions.

Based on these findings, the approximate lethal dose of the A6K/BSH boron drug is presumed to exceed 600 mg/kg (BSH equivalent) under the conditions of this study. Previous investigations have demonstrated that the A6K/BSH boron drug exhibits excellent tumor selectivity and exceptional intracellular boron uptake in vitro without apparent cytotoxicity. Tumor-specific accumulation following tail vein administration has also been reported in mouse brain tumor models [13]. These findings, together with the absence of short-term systemic toxicity observed in the present single-dose study, suggest that this formulation may function as a boron delivery agent capable of enhancing tumor boron delivery without increasing toxicity to normal tissues.

In contrast, previously published toxicity studies on BSH alone have reported mortality and organ damage in high-dose groups [22]. In the present study, however, no mortality or organ toxicity had been observed following intraperitoneal administration of the A6K/BSH boron drug at the same BSH-equivalent dose of 600 mg/kg. Several factors may account for this discrepancy.

First, differences in the route of administration should be considered. Prior toxicity studies on BSH alone were primarily conducted using intravenous administration. Rapid intravenous injection of a high-concentration formulation may produce transiently elevated plasma concentrations, potentially causing abrupt alterations in hemodynamics or organ perfusion. In contrast, the intraperitoneal route employed in the current study generally results in more gradual absorption and lower peak plasma concentrations, which may attenuate acute toxicity. Although intraperitoneal administration has been widely used in preclinical research as an alternative to intravenous administration given that it allows for systemic exposure relatively comparable to the intravenous route in terms of pharmacological effects, it does not necessarily constitute a complete substitute, considering that differences in pharmacokinetics and tissue distribution have been reported [31,32].

Second, differences in formulation and in vivo distribution may have contributed to discrepancies between the current study and those published previously. Research shows that the A6K/BSH boron drug consists of BSH encapsulated within the A6K peptide-based drug delivery system [13]. This structural configuration may suppress the concentration of free BSH in the circulation or peritoneal cavity, thereby reducing acute exposure of normal organs. However, given that the present study failed to measure plasma concentration profiles and organ boron levels, the in vivo biodistribution and elimination pathways remain to be elucidated.

It is important to recognize that these factors are not mutually exclusive, and each may have independently contributed to the reduced toxicity observed in the present study. Definitive delineation of the relative contribution of each factor will require future studies employing intravenous administration of the A6K/BSH boron drug with comprehensive pharmacokinetic analyses, including measurement of plasma and tissue boron concentrations. Accordingly, the present study should not be interpreted as demonstrating lower toxicity of the A6K/BSH boron drug compared with BSH alone. Rather, it shows that single-dose intraperitoneal administration of the present formulation up to 600 mg/kg (BSH-equivalent) was not associated with lethality or overt systemic toxicity in BALB/c mice.

Several limitations of this study should be acknowledged. First, the evaluation was limited to a single-dose toxicity study. Hence, the effects of multiple administrations or long-term toxicity remain unknown. Second, pharmacokinetic parameters, including plasma concentration–time profiles and organ-specific boron concentrations, were not determined, which precludes direct comparison of systemic boron exposure between this study and previous reports using different formulations or administration routes. Third, given that the drug was administered via the intraperitoneal route owing to the viscosity constraints of the high-concentration formulation, the safety of the A6K/BSH boron drug under clinically relevant intravenous conditions was not directly assessed. However, this limitation is specific to the high-concentration research-grade formulation used in the present study and does not necessarily preclude intravenous administration under clinical conditions. In clinical BNCT practice, boron agents are typically administered by slow intravenous infusion at appropriately diluted concentrations [9,10], under which the viscosity-related issues observed in the present formulation may be reduced. Therefore, future pharmaceutical development of the A6K/BSH boron drug should include formulation optimization for intravenous compatibility, including evaluation of particle size, filterability, and infusion stability [10,11]. Fourth, nanostructural characteristics such as particle size distribution, aggregation state, and stability were not re-evaluated in the present study, although these properties have been previously reported for the same formulation. Reassessment of these parameters under the current experimental conditions would further strengthen the interpretation of the results.

Despite these limitations, the current study demonstrated that a single intraperitoneal administration of the A6K/BSH boron drug did not induce evident acute toxicity at doses of up to 600 mg/kg (BSH-equivalent). These findings represent an important initial step in the safety evaluation of this formulation as a boron delivery agent for BNCT. Future studies should include systematic single- and repeated-dose toxicity assessments under intravenous administration, comprehensive pharmacokinetic analyses of blood and tissue boron concentrations, and integrated evaluations of efficacy and safety in tumor-bearing models to further clarify the safety profile of the A6K/BSH boron drug. In particular, given that the reduced toxicity observed relative to previous BSH-only studies [22] may reflect contributions from both the intraperitoneal administration route and the A6K-based formulation, future intravenous studies with concurrent pharmacokinetic monitoring will be essential to distinguish these factors and to establish the safety profile of the A6K/BSH boron drug under conditions more closely approximating clinical use.

## 5. Conclusions

The present study demonstrated that a single intraperitoneal administration of self-assembling A6K/BSH peptide nanotubes (A6K/BSH boron drug) up to 600 mg/kg (BSH-equivalent) did not induce mortality, apparent clinical signs, or gross organ toxicity in mice. Mild treatment-related histopathological changes in the liver were observed; however, these findings were not dose-dependent and were considered indicative of a non-adverse adaptive metabolic response rather than overt hepatotoxicity. These findings provide an important initial assessment of the safety of the A6K/BSH boron drug as a boron delivery agent for BNCT. Future studies involving intravenous administration, repeated dosing, pharmacokinetic analyses, and integrated efficacy and safety evaluations in tumor models are warranted to further establish its preclinical safety profile.

## Figures and Tables

**Table 1:** Amount of BSH contained according to the concentration of the A6K/BSH boron drug.

Concentration of the A6K/BSH Boron Drug (A6K mM:BSH mM)	Amount of BSH Contained (mg/1000 μL A6K/BSH Boron Drug)
2:20	4.2
5:50	10.5
10:100	21.0
20:200	42.0

BSH, sodium borocaptate.

**Table 2:** Dosage and administration volume in the experimental groups.

Group	BSH Dosage (mg/kg *)	Concentration of the Used A6K/BSH Boron Drug (A6K mM:BSH mM)	A6K/BSH Boron Drug Administration Volume of (mL/kg *)
G0	0	–	–
G1	30	2:20	7.14
G2	100	5:50	9.52
G3	300	10:100	14.29
G4	600	20:200	14.29

* kg, mouse body weight. BSH, sodium borocaptate.

**Table 3:** Effects of the A6K/BSH boron drug on mortality and clinical signs.

Group	BSH Dosage (mg/kg *)	No. of Animals (Male/Female)	Mortality (Male/Female)	Clinical Signs
G0	0	3/3	0/0	No abnormality detected
G1	30	3/3	0/0	No abnormality detected
G2	100	3/3	0/0	No abnormality detected
G3	300	3/3	0/0	No abnormality detected
G4	600	3/3	0/0	No abnormality detected

* kg, mouse body weight. BSH, sodium borocaptate.

**Table 4:** Necropsy findings in a single-dose intraperitoneal toxicity study of the A6K/BSH boron drug in mice.

Group	BSH Dosage (mg/kg *)	No. of Animals (Male/Female)	Organ Necropsy Findings	Type of Sacrifice
G0	0	3/3	No abnormality detected	Scheduled
G1	30	3/3	No abnormality detected	Scheduled
G2	100	3/3	No abnormality detected	Scheduled
G3	300	3/3	No abnormality detected	Scheduled
G4	600	3/3	No abnormality detected	Scheduled

* kg, mouse body weight. BSH, sodium borocaptate.

**Table 5:** Histopathological findings in a single-dose intraperitoneal toxicity study of the A6K/BSH boron drug in mice.

Organ	Findings	Group/BSH Dosage (mg/kg *)				
		G0/0	G1/30	G2/100	G3/300	G4/600
Liver	Hepatocellular hypertrophy	^#^ 0/6	6/6	6/6	6/6	6/6
	Granular generation	0/6	6/6	6/6	6/6	6/6

* kg, mouse body weight. ^#^ Number of affected mice/total mice (males and females). BSH, sodium borocaptate.

## Data Availability

The data presented in this study are available on request from the corresponding author due to ethical reasons.

## Supplementary Materials

The following supporting information can be downloaded at: https://www.mdpi.com/article/10.3390/cancers18091382/s1, Table S1: Histopathological findings (major organs) in the single-dose intraperitoneal toxicity study of the A6K/BSH boron drug in mice. All findings were recorded as 0/6 affected mice in every group (n = 3 males + 3 females per group).

## Abbreviations

The following abbreviations are used in this manuscript:

BNCT	Boron neutron capture therapy
ICI	Immune checkpoint inhibitors
