# Supplementary material for: Single-Dose Toxicity Study of Self-Assembling A6K/Sodium Borocaptate (BSH) Peptide Nanotubes as a New Boron Delivery Agent for Boron Neutron Capture Therapy (BNCT) in Mice
Source: Cancers (Basel). 2026 Apr 27;18(9):1382. doi: 10.3390/cancers18091382 (PMC13162931; doi:10.3390/cancers18091382)
Supplement: Supplementary file 1 [file cancers-18-01382-s001.zip › cancers-4258990-supplementary.pdf]

Supplementary Materials

Supplementary Table S1. Histopathological findings (major organs) in the single-dose intraperitoneal toxicity study of the A6K/BSH boron drug in mice. All findings were recorded as 0/6 affected mice in every group (n = 3 males + 3 females per group).

Organ	Findings	G0/0	G1/30	G2/100	G3/300	G4/600
Liver	Single cell necrosis	0/6	0/6	0/6	0/6	0/6
	Apoptosis	0/6	0/6	0/6	0/6	0/6
	Focal necrosis	0/6	0/6	0/6	0/6	0/6
	Fibrosis	0/6	0/6	0/6	0/6	0/6
Heart	Necrosis	0/6	0/6	0/6	0/6	0/6
	Fibrosis	0/6	0/6	0/6	0/6	0/6
	Inflammation	0/6	0/6	0/6	0/6	0/6
Lung	Desquamation, terminal bronchiole	0/6	0/6	0/6	0/6	0/6
	Necrosis, terminal bronchiole	0/6	0/6	0/6	0/6	0/6
	Fibrosis, alveolus	0/6	0/6	0/6	0/6	0/6
	Inflammation	0/6	0/6	0/6	0/6	0/6
Pancreas	Inflammatory cell infiltration, islet formation	0/6	0/6	0/6	0/6	0/6
	Atrophy, islet	0/6	0/6	0/6	0/6	0/6
	Vacuolation, islet	0/6	0/6	0/6	0/6	0/6
	Amyloid deposition, islet formation	0/6	0/6	0/6	0/6	0/6
	Fibrosis, islet	0/6	0/6	0/6	0/6	0/6
	Vacuolation, acinus	0/6	0/6	0/6	0/6	0/6
	Fatty change, acinus	0/6	0/6	0/6	0/6	0/6
	Liquefactive necrosis, acinus	0/6	0/6	0/6	0/6	0/6
	Apoptosis, acinus	0/6	0/6	0/6	0/6	0/6
	Fibrosis, acinus	0/6	0/6	0/6	0/6	0/6
Kidney	Mesangial hyperplasia, glomerulus	0/6	0/6	0/6	0/6	0/6
	Vacuolation, glomerulus	0/6	0/6	0/6	0/6	0/6
	Hyaline droplet degeneration, glomerulus	0/6	0/6	0/6	0/6	0/6
	Atrophy, glomerulus	0/6	0/6	0/6	0/6	0/6
	Necrosis, tubule	0/6	0/6	0/6	0/6	0/6
	Regeneration, tubule	0/6	0/6	0/6	0/6	0/6
	Atrophy, tubule	0/6	0/6	0/6	0/6	0/6
	Dilatation, tubule	0/6	0/6	0/6	0/6	0/6
	Vacuolation, tubule	0/6	0/6	0/6	0/6	0/6
	Necrosis, papillary duct	0/6	0/6	0/6	0/6	0/6

	Lymphoid infiltration, stroma	0/6	0/6	0/6	0/6	0/6
	Fibrosis, stroma	0/6	0/6	0/6	0/6	0/6
	Pyelitis	0/6	0/6	0/6	0/6	0/6
Bone marrow	Absence of megakaryocytes, erythroid islands, or granulocytic precursors	0/6	0/6	0/6	0/6	0/6
	Increased/reduced marrow cellularity	0/6	0/6	0/6	0/6	0/6
	Fibrosis	0/6	0/6	0/6	0/6	0/6

Note: G0/0 represents the control group. G1/30, G2/100, G3/300, and G4/600 represent groups administered the A6K/BSH boron drug at BSH-equivalent doses of 30, 100, 300, and 600 mg/kg, respectively. Counts indicate the number of affected mice / total mice evaluated.
